# Supplementary material for: Evaluation of Three-Dimensional Bioprinted Human Cartilage Powder Combined with Micronized Subcutaneous Adipose Tissues for the Repair of Osteochondral Defects in Beagle Dogs
Source: Int J Mol Sci. 2022 Mar 1;23(5):2743. doi: 10.3390/ijms23052743 (PMC8910734; doi:10.3390/ijms23052743)
Supplement: Supplementary file 1 [file ijms-23-02743-s001.zip › ijms-1619830-supplementary.pdf]

Table S1. Compressive analysis

		Stiffness (N/mm^2)	st dev	Relax_coeff (MPa/sec)	st dev	Final Stress Relax (N)	st dev
	Normal	15.12	6.78	-3.16E-05	5.92E-05	0.09	0.06
20 week (Right)	Defect	28.14	16.36	-2.91E-05	5.74E-06	0.39	0.23
	LCCM	10.97	6.02	-7.64E-06	3.06E-06	0.21	0.15
	MA-ECM	8.86	3.33	-6.64E-06	-	0.05	-
	LCCM/ MA-ECM	12.84	3.85	-1.70E-05	1.49E-05	0.28	0.16
32 week (Left)	Defect	8.28	9.35	-9.98E-05	0.000121	0.17	0.10
	LCCM	12.92	4.16	-1.55E-06	7.82E-06	0.20	0.15
	MA-ECM	13.49	4.80	-6.66E-06	1.27E-05	0.28	0.17
	LCCM/ MA-ECM	23.25	17.91	-2.77E-05	1.69E-05	0.36	0.20

Table S2. Guideline for MOCART 2 knee scoring

MOCART 2 Knee Score	Points
**Volume fill of cartilage defect**	
Complete filling or minor hypertrophy: 100% to 150% filling of total defect volume	20
Major hypertrophy ≥150% Or 75% to 99% filling of total defect volume	15
50% to 74% filling of total defect volume	10
25% to 49% filling of total defect volume	5
<25% filling of total defect volume Or complete delamination in situ	0
**Integration into adjacent cartilage**	
Complete integration	15
Split-like defect at repair tissue and native cartilage interface ≤2 mm	10
Defect at repair tissue and native cartilage interface >2 mm, but <50% of repair tissue length	5
Defect at repair tissue and native cartilage interface ≥50% of repair tissue length	0
**Surface of the repair tissue**	
Surface intact	10
Surface irregular <50% of repair tissue diameter	5
Surface irregular ≥50% of repair tissue diameter	0
**Structure of the repair tissue**	
Homogeneous	10
Inhomogeneous	0
**Signal intensity of the repair tissue**	
Normal	15
Minor abnormal—minor hyperintense (5_2a) Or minor hypointense	10
Severely abnormal—almost fluid like (5_3a) Or close to subchondral plate signal	0
**Bony defect or bony overgrowth**	
No bony defect or bony overgrowth	10
Bony defect: depth < thickness of adjacent cartilage Or overgrowth <50% of adjacent cartilage	5
Bony defect: depth ≥ thickness of adjacent cartilage Or overgrowth ≥50% of adjacent cartilage (6_2b)	0
**Subchondral changes**	
No major subchondral changes	20
Minor edema-like marrow signal—maximum diameter <50% of repair tissue diameter	15
Severe edema-like marrow signal—maximum diameter ≥50% of repair tissue diameter	10
Subchondral cyst ≥5 mm in longest diameter Or osteonecrosis-like signal	0
**Effusion**	
No effusion	5
Effusion	0
**Total**	105

Table S3. Guideline for ICRS visual histological assessment scale

ICRS Visual Histological Assessment	Score
**Surface**	
Smooth/continuous	3
Discontinuities/irregularities	0
**Matrix**	
Hyaline	3
Mixture: hyaline/fibrocartilage	2
Fibrocartilage	1
Fibrous tissue	0
**Cell distribution**	
Columnar	3
Mixed/columnar-clusters	2
Clusters	1
Individual cells/disorganized	0
**Cell population viability**	
Predominantly viable	3
Partially viable	1
<10% viable	0
**Subchondral Bone**	
Normal	3
Increased remodeling	2
Bone necrosis/granulation tissue	1
Detached/fracture/callus at base	0
**Cartilage mineralization (calcified cartilage)**	
Normal	3
Abnormal/inappropriate location	0

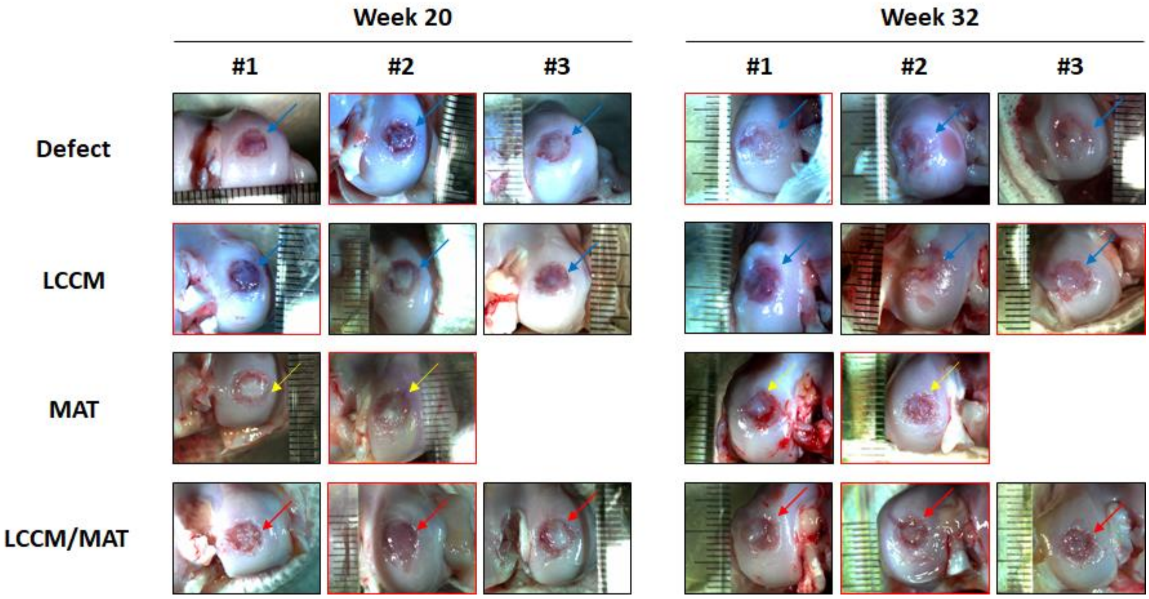

Figure S1. Gross appearance of the femoral cartilages.
The appearance of the cartilages after bi-lateral 3D-printed matrix implantation procedure performed on 12 male beagle dogs. Firstly, on the left knee followed by 12 weeks later by the contralateral knee. The arrows indicate circular chondral defect area with a diameter of 6 mm in each group. Note that red arrows in LCCM/MAT groups showed homogenously recovered defects compared to heterogeneously composed (blue arrows) and partially recovered (yellow arrows) ones. The picture marked with a red border is the representative image of each group.

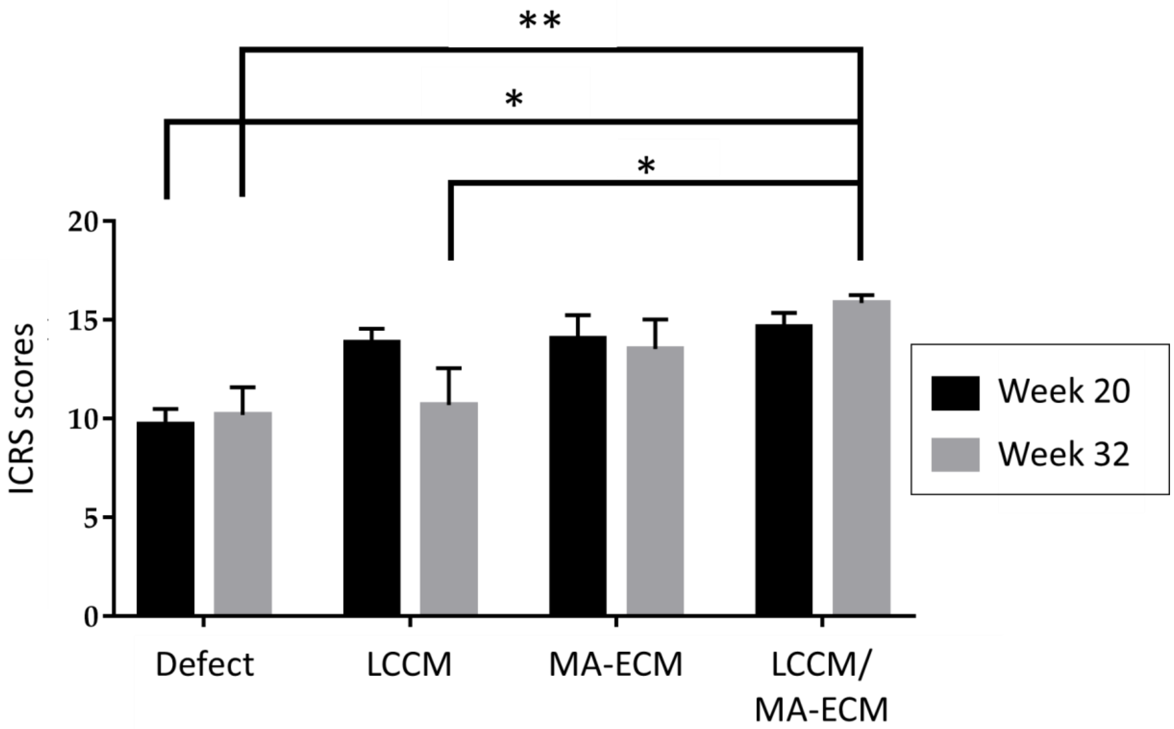

Figure S2. Visual histopathological quantitative macroscopic evaluation using ICRS criteria. All raw data were analyzed with two-way ANOVA followed by Tukey's multiple comparison tests (n = 3). **p* < 0.05, ***p* < 0.01, ****p* < 0.001
